# Improving feedback on junior doctors’ prescribing errors: mixed-methods evaluation of a quality improvement project

**DOI:** 10.1136/bmjqs-2015-004717

**Published:** 2016-04-04

**Authors:** Matthew Reynolds, Seetal Jheeta, Jonathan Benn, Inderjit Sanghera, Ann Jacklin, Digby Ingle, Bryony Dean Franklin

**Affiliations:** 1 Centre for Medication Safety and Service Quality, Imperial College Healthcare NHS Trust, London, UK; 2 Centre for Patient Safety and Service Quality, Imperial College London, London, UK; 3 Department of Pharmacy, London North West Healthcare NHS Trust, London, UK; 4 Royal College of Pathologists, London, UK; 5 Department of Practice and Policy, UCL School of Pharmacy, London, UK

**Keywords:** Audit and feedback, Quality improvement, Medical education, Medication safety

## Abstract

**Background:**

Prescribing errors occur in up to 15% of UK inpatient medication orders. However, junior doctors report insufficient feedback on errors. A barrier preventing feedback is that individual prescribers often cannot be clearly identified on prescribing documentation.

**Aim:**

To reduce prescribing errors in a UK hospital by improving feedback on prescribing errors.

**Interventions:**

We developed three linked interventions using plan–do–study–act cycles: (1) name stamps for junior doctors who were encouraged to stamp or write their name clearly when prescribing; (2) principles of effective feedback to support pharmacists to provide feedback to doctors on individual prescribing errors and (3) fortnightly prescribing advice emails that addressed a common and/or serious error.

**Implementation and evaluation:**

Interventions were introduced at one hospital site in August 2013 with a second acting as control. Process measures included the percentage of inpatient medication orders for which junior doctors stated their name. Outcome measures were junior doctors' and pharmacists' perceptions of current feedback provision (evaluated using quantitative pre-questionnaires and post-questionnaires and qualitative focus groups) and the prevalence of erroneous medication orders written by junior doctors between August and December 2013.

**Results:**

The percentage of medication orders for which junior doctors stated their name increased from about 10% to 50%. Questionnaire responses revealed a significant improvement in pharmacists' perceptions but no significant change for doctors. Focus group findings suggested increased doctor engagement with safe prescribing. Interrupted time series analysis showed no difference in weekly prescribing error rates between baseline and intervention periods, compared with the control site.

**Conclusion:**

Findings suggest improved experiences around feedback. However, attempts to produce a measurable reduction in prescribing errors are likely to need a multifaceted approach of which feedback should form part.

## Introduction

Prescribing errors occur in 1%–15% of medication orders written for UK hospital inpatients, resulting in harm to an estimated 1%–2%.^1^ Local studies suggest a similar prevalence of error.^2^ While there have been relatively few studies of interventions to reduce them,^3^ a common theme in UK studies of the causes of prescribing errors^2^ ^4^ ^5^ is that hospital doctors are often unaware of their errors. Provision of explicit feedback to junior doctors and encouragement of help-seeking behaviours have been recommended to address this.^2^ ^4–6^ In the improvement and implementation science literature, feedback has been widely studied as a means of improving outcomes and changing professional behaviour.^7^ Research suggests that a number of characteristics are important for effective feedback, including perceived usefulness, credibility, intensity, timeliness, relevance and the presence of linked quality improvement mechanisms.^8–12^

**Figure 1:**
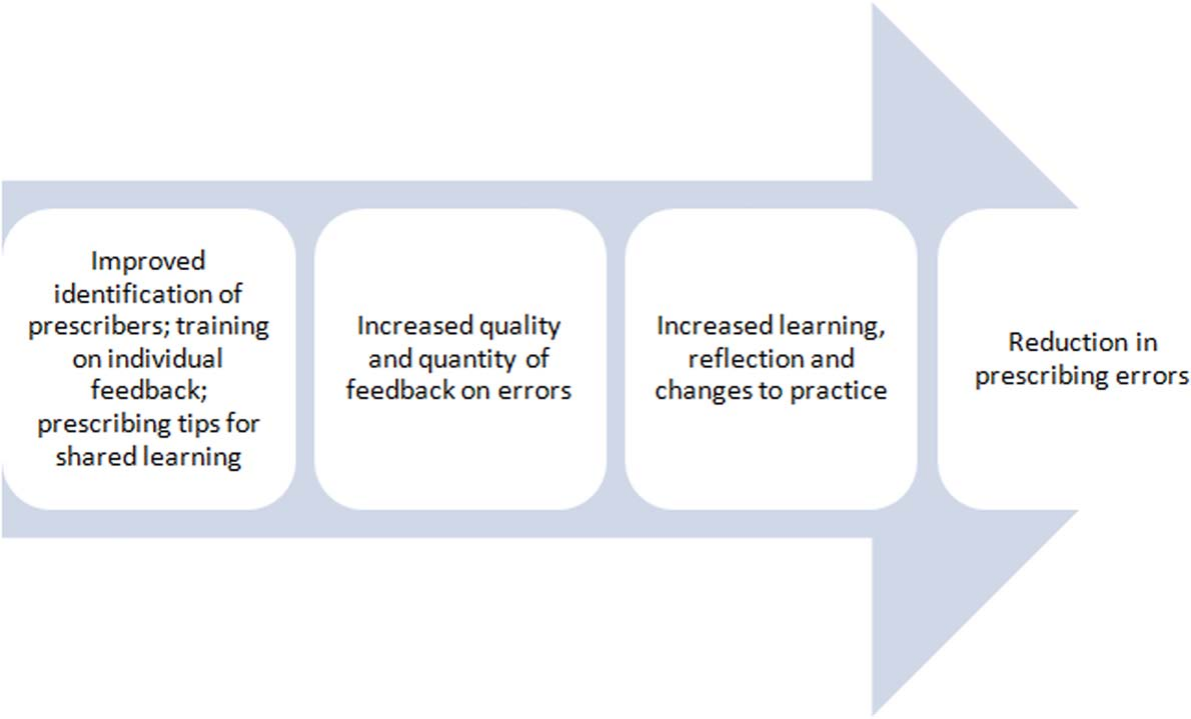
Logic model: basic high-level model depicting the planned inputs and intended results.^16^

Previous work within our organisation confirmed that lack of feedback was a problem locally.^2^ We therefore wanted to conduct a quality improvement study aimed at reducing prescribing errors through provision of feedback. We undertook pilot work providing feedback to teams of doctors^13^ and ascertained the acceptability of pharmacists providing more formal feedback to junior doctors on an individual basis.^14^ However, a barrier was that individual prescribers could only be identified for about 10% of individual inpatient medication orders.^15^ Our basic logic model^16^ (figure 1) was that improved prescriber identification and better provision of feedback^17^ on prescribing errors would facilitate learning, reflection and changes to practice. This would in turn reduce prescribing errors. Drawing on prior research in the area of audit and feedback, our theory of change was that the implementation of timely, relevant feedback to prescribers would effect changes in professional behaviour and reduce prescribing errors.^8–12^ This paper describes the development, testing and evaluation of our approach, structured according to the SQUIRE guidelines.^18^ Our objectives were to improve prescriber identification on medication orders in order to facilitate individual feedback and to provide effective feedback to prescribers at both the individual level and group level with the aim of reducing prescribing errors.

## Methods

### Setting

The study took place at two large teaching hospitals within the same UK hospital organisation. We focused on junior doctors, specifically foundation year 1 doctors (those in their first year post-medical school). This group of doctors do a large proportion of prescribing,^5^ having a relatively high prescribing error rate,^5^ and good prescribing habits learnt early will hopefully be continued throughout their career. Online supplementary appendix S1 explains UK medical staff grades. The interventions were developed and piloted with existing junior doctors and pharmacists at the intervention hospital (site 1) between February and July 2013. The interventions were then implemented at this site concurrently with the new cohort of junior doctors starting in August 2013. A second hospital (site 2) acted as control for selected aspects of the evaluation. At any one time there were 30–34 junior doctors at site 1, mainly based on 12 wards, and 43–45 at site 2, again across 12 wards. Both sites used the same preformatted paper medication charts on which medication orders were handwritten for inpatients and electronic prescriptions at discharge. Junior doctors generally rotated between specialties within the same hospital every 4 months.

10.1136/bmjqs-2015-004717.supp1 Supplementary appendix 1

Pharmacists provided a typical UK ward pharmacy service, with wards generally visited by a pharmacist for 1–3 h each weekday. Pharmacists checked inpatient medication orders and discharge prescriptions to ensure that they were clear, legal and clinically appropriate. Standard practice was that any prescribing errors identified were rectified following discussion with a prescriber, if necessary. If the original prescriber could not easily be identified and/or contacted, any available prescriber was asked to rectify the error.

### Developing the interventions

Previous research suggests specific mechanisms that maximise the effectiveness of feedback and its capacity to elicit behavioural change. Feedback is most effective when provided more than once, using both verbal and written formats and including both explicit targets and an action plan.^7^ ^19^ To develop interventions that incorporated this best practice, a panel of six existing junior doctors was recruited to contribute to initial development and testing using plan–do–study–act (PDSA) cycles between February and July 2013. We focused on three linked interventions: improving prescriber identification, developing an agreed approach for pharmacists to use when providing feedback on individual prescribing errors and a method of sharing learning among all junior doctors following common or serious errors.

#### Intervention 1: prescriber identification

To facilitate prescriber identification on inpatient medication charts, we used a multifaceted approach. Junior doctors were given personalised name stamps and were reminded to write or stamp their name whenever prescribing on paper medication charts. They were emailed fortnightly run charts showing their compliance with prescriber identification as a group, established by a member of the project team using a signature log created for the project. Following suggestions from junior doctors in early PDSA cycles, we subsequently modified the emails to include comparison among the three main clinical specialities, which introduced an element of competition. The inpatient medication chart used on both hospital sites was also modified to clearly specify that the prescriber's surname was required alongside their signature and pager number.

#### Intervention 2: individual feedback

We worked with our panel of six junior doctors to agree some principles of effective feedback plus a set of phrases that they felt would clearly communicate that an error had been made in a non-threatening manner (box 1). Training sessions were held with pharmacists to explain why and how to provide feedback on prescribing errors, incorporating quotes from junior doctors to highlight the importance of appropriate feedback. Where possible, a junior doctor representative attended to explain first hand why feedback was valued. The principles were also incorporated into local clinical pharmacy standard operating procedures.

Box 1 **Principles of effective feedback on individuals' prescribing errors and suggested phrases to use, developed from the literature^8–10^ and our panel of six junior doctors**

Principles of effective feedback

Feedback should:

- be as soon as possible after the event;
- ensure the prescriber is aware that an error has been made;
- discuss possible solutions;
- highlight any relevant prescribing resources (eg, clinical guidelines) and
- be non-judgemental and blame-free.

Suggested phrases

- ‘I want to highlight to you that there's an error on this drug chart. The correct way to prescribe it is…’.
- ‘This dose is incorrect for this patient: it should be……here's where you find the protocol’.

#### Intervention 3: shared learning

To support shared learning, ‘good prescribing tip’ emails were developed and sent fortnightly to site 1 junior doctors and pharmacists. Topics were based on local incident reports or suggestions from pharmacy or medical staff. Each prescribing tip (see online supplementary appendix S2) focused on one specific serious/common error and provided evidence-based solutions, aligned with local guidelines. PDSA cycles were used to develop materials perceived as relevant by the target audience and visually appealing on both desktops and smartphones.

10.1136/bmjqs-2015-004717.supp2 Supplementary appendix 2

### Evaluation of the interventions

We used a mixed-methods approach with quantitative and qualitative elements to evaluate changes in key process and outcome measures as well as exploring the experiences of the prescribers and pharmacists involved.

### Process measures

#### Prescriber identification

A signature log was created for all junior doctors; this was used by a member of the project team to conduct a weekly audit on the 12 wards on each site where junior doctors were based. The first 10 available medication charts on each ward were examined for prescriber identification (presence of a signature, plus either a handwritten or stamped name) for junior doctors' medication orders. Prescriber identification rates were compared pre-intervention and post-intervention using a χ^2^ test.

#### Pharmacists' provision of individual feedback

We added three extra criteria to the standard set of 43 criteria used to assess pharmacists during routine peer-reviewed ward visits. Peer-reviewed visits were routinely conducted by senior clinical pharmacists employed at the study hospitals based on a validated approach^20^; the three extra criteria relating to feedback were added only at site 1. For any prescribing errors identified, these new criteria established whether pharmacists attempted to contact the initial prescriber, whether they gave appropriate feedback and whether they highlighted any relevant prescribing resources. Each criterion was assessed on a 4-point Likert scale (always, mostly, sometimes, never).^20^

### Outcome measures

#### Junior doctors' and pharmacists' perceptions of feedback

Evaluative questionnaires (see online supplementary appendix S3) were developed for both junior doctors and pharmacists, including items based on characteristics of effective feedback: perceived usefulness, credibility, intensity, timeliness, relevance and accuracy of feedback.^8–10^ All measures were reported on an 8-point scale with higher values representing more positive responses. Items were additionally combined into a mean overall feedback effectiveness scale score (Cronbach's α 0.904 for pharmacists and 0.853 for junior doctors). The questionnaire was administered at site 1 during spring 2013 (pre-intervention) and again in spring 2014 (approximately 9 months post-intervention) to a different cohort of junior doctors at the same point in their training year, and to all pharmacists employed at each time point. Mean scores were compared pre-intervention and post-intervention using independent samples t-tests, with p<0.05 indicating statistical significance for the overall feedback effectiveness scales and p<0.01 for each of the individual items (to account for multiple testing).

10.1136/bmjqs-2015-004717.supp3 Supplementary appendix 3

Two focus groups were conducted at site 1, one for junior doctors and one for pharmacists, to explore views on our interventions in more detail. Four main topics were explored: prescriber identification and name stamps, prescriber identification run charts, individual feedback of prescribing errors and prescribing tip emails. Unintended consequences of the interventions were also explored. Discussions were recorded and professionally transcribed. We used thematic analysis^21^ with a deductive approach using an a priori framework of the perceived advantages, disadvantages, facilitators and barriers of each intervention.

#### Prescribing error prevalence

We studied the prevalence of erroneous medication orders written by junior doctors at site 1 (intervention) and site 2 (control) using identical methods on each site. Following verbal and written briefings, including our definition of a prescribing error,^22^ ward pharmacists collected data once weekly from August to December 2013 on any prescribing errors identified in the first 10 junior doctors' medication orders encountered, using the signature logs produced by the project team. Errors were classified based on previous work^2^; each medication order could have more than one error. We used interrupted time series analysis to test for an effect of our intervention on weekly prescribing error rate at the intervention site, controlling for baseline variation in error rates and longitudinal variation in error rates at the control site.^23^ The time series components of the model were fitted as segmented regression with parameters representing baseline trend, intervention onset and the post-intervention change in trend. A significant reduction in weekly error rate and/or trend in error rate, as indicated by p<0.05 for the value of *t* associated with each parameter coefficient, would indicate a positive effect of the intervention.

**Figure 2:**
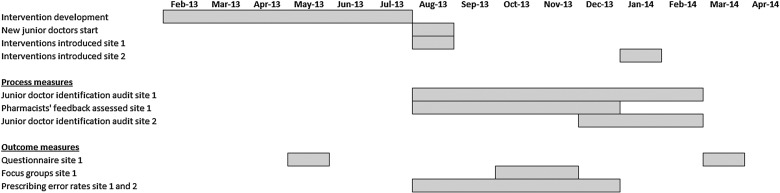
Project timeline.

**Figure 3:**
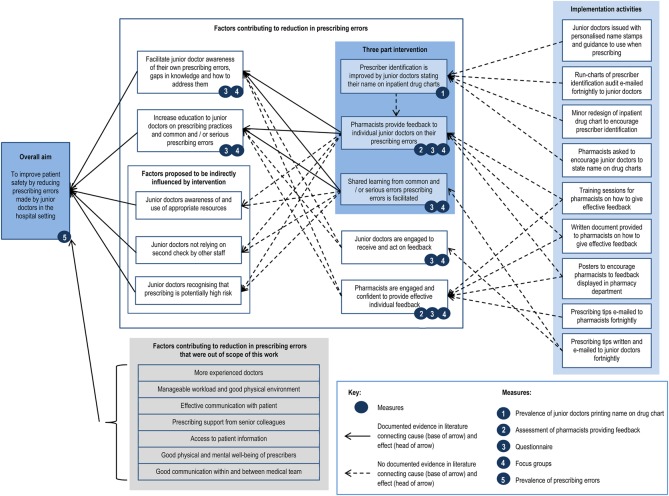
Prescribing improvement action–effect diagram.

Figure 2 presents the study timeline. An action–effect diagram^24^ is presented in figure 3.

### Ethical issues

The study was approved locally as a service evaluation; NHS ethics approval was not required. Data could not be attributed to individual patients or staff. Focus group participants provided written informed consent.

## Results

### Course of the intervention

Following a series of four PDSA cycles, name stamps and supporting information were distributed to all junior doctors at site 1 during their induction in August 2013, before they started patient care. Pharmacists were briefed on providing feedback during July and August 2013. We estimate that around half of the department's pharmacists attended a face-to-face training session. Through regular publicity, including presentations at pharmacy staff meetings, posters and emails, we are confident that all pharmacists were aware of our aims. Following three PDSA cycles, prescribing tip emails were produced every fortnight starting August 2013. All interventions were subsequently rolled out during January 2014 to also include all junior doctors and pharmacists at site 2.

### Process measures

#### Prescriber identification

At site 1, 11 374 medication orders were examined over 29 weeks post-intervention (mean 392 per week). The prescriber's name was present for 5935 (52.2%), of which 3617 (60.9%) were stamped names. No comparative baseline data were available for site 1 as the interventions were introduced contemporaneously with the junior doctors starting employment.

At site 2, where the interventions were introduced later, 789 medication orders were examined at baseline over 3 weeks (mean 263 per week) and 2332 during six post-intervention weeks (mean 389 per week). Pre-intervention, the prescriber's name was specified on 48 of 789 (6.1%) medication orders; this increased to 860 of 2332 (36.9%) post-intervention (p<0.0001; χ^2^ test), of which 501 (58.3%) were stamped.

#### Pharmacists' provision of individual feedback

Senior clinical pharmacists recorded data for five peer-reviewed ward visits that took place at site 1 between August and December 2013. In three of the five visits, pharmacists ‘always’ attempted to contact the original prescriber and provided feedback in a professional manner and in the remaining two visits this was done ‘mostly’ as assessed by the peer reviewer. Signposting to suitable resources or solutions was done less consistently.

### Outcome measures

#### Junior doctors' and pharmacists' perceptions of feedback

At baseline (Spring 2013), we received questionnaire responses from 25 of 37 (68%) site 1 pharmacists and 26 of 34 (76%) junior doctors. At the second time point (Spring 2014), we received 18 of 32 (56%) pharmacist responses and 21 of 30 (70%) from junior doctors. For the overall feedback effectiveness scale scores, the mean score for junior doctors increased slightly post-intervention (mean at baseline=6.15, SD=0.74; mean post-intervention=6.23; SD=0.86), but was not statistically significant (p=0.74; unpaired t-test). The mean score for pharmacists improved significantly post-intervention (mean at baseline=5.26, SD=0.88; mean post-intervention=5.84, SD=0.80; p=0.03). For the junior doctors' responses, improvement was observed in the mean response for five individual items, although none reached statistical significance (see online supplementary appendix S4). For pharmacists, there was improvement in 14 items. Two reached statistical significance at p<0.01: perceptions of the accuracy of feedback delivered to junior doctors (item 15; p=0.003) and perceptions of whether junior doctors found the data to be trustworthy (item 16; p=0.007), with improvements in mean scores of 1.4 and 0.87, respectively.

10.1136/bmjqs-2015-004717.supp4 Supplementary appendix 4

A total of 14 doctors (42% of 33 at site 1) attended the junior doctors' focus group in October 2013, all of whom participated in the discussion. Participants expressed positive views and experiences of receiving feedback and reported increased understanding of the importance of ensuring their identity is known when prescribing. Prescriber identification run charts and prescribing tips were well received and thought to be useful, although some felt fortnightly run charts were too frequent. Quotes are presented in table 1.

**Table 1:** Quotes from junior doctors' focus group

Intervention	Quotes
Prescriber identification	I think the fact that we'd been given the stamps highlighted the fact more than anything that we should be putting our names on the paperwork. Now even when I don't have my stamp I think, oh, I'd better write my name because I don't have my stamp on me. I find using the stamp makes me take a lot more ownership of it, I think, do I really know what I'm doing? I'm putting my name to that, like George Foreman grill, it'd better work.
Individual feedback	[The prescription] would need to be changed [because of] patient safety, and that can be done by anyone on the team, but I'd like to know personally that I'd made a mistake. …[I]t's hard to say what it would be like without [receiving feedback], it makes you feel safe because it feels like every error you may be making is being checked by somebody and that they are then feeding back. So it feels like I'm making less errors now because I've learnt from the errors I've made.
Shared learning	[The prescribing tips are] good because, there's often a picture of a drug chart so you can look at it quite quickly and…You don't have to read a lot of text, you can just look and read, oh, I can see just a gap, that … if you're just quickly checking it because you don't have time.

Four pharmacists attended the pharmacists' focus group in November 2013. Pharmacists felt that feedback was generally well received, although they felt uncomfortable referring explicitly to ‘errors’ and seemed protective of relationships with their junior doctor colleagues. They preferred to use the terms ‘mistake’ or ‘incorrect’. They felt that pharmacists were providing more individualised feedback due to increased awareness of the benefits and had additionally used the prescribing tips to facilitate discussions. No unintended negative consequences were identified by either group. An unintended positive consequence was junior doctors using the name stamps in the written health records as well as for prescribing.

#### Prescribing error prevalence

We identified 390 errors in 367 (15.2%) of 2410 medication orders at site 1 and 391 errors in 368 (15.1%) of 2432 medication orders at site 2 (table 2). Overall, the most common error types (see online supplementary appendix S5) were ‘incorrect dose’ (175 of 781 errors; 22%) and ‘medication omitted when clinically indicated’ (172; 22%), usually involving omission of patients' usual medication following hospital admission.

**Table 2:** Error rates for intervention and control sites

Site	Pre-intervention erroneous medication orders (% of all medication orders)	Post-intervention erroneous medication orders (% of all medication orders)	Overall erroneous medication orders (% of all medication orders)
1 (intervention)	92 of 620 (14.8)	275 of 1790 (15.4)	367 of 2410 (15.2)
2 (control)	35 of 276 (12.7)	333 of 2156 (15.5)	368 of 2432 (15.1)

10.1136/bmjqs-2015-004717.supp5 Supplementary appendix 5

Over the course of the project, the mean weekly prescribing error rate at both sites increased between baseline and follow-up periods. When an interrupted time series model was fitted to examine the change in mean error rate, while controlling for baseline variation and compared with the control site, no significant effect of the intervention at site 1 was observed. The overall effect of intervention implementation (ie, the unique effect associated with the intervention while holding baseline trend and control constant) was a non-significant reduction in the error rate of 4% (β=−4.045; t=−0.638; p=0.532). Similarly the estimated trend in error rate was a non-significant decrease of 4% post-intervention (β=−4.440; t=−1.097; p=0.288).

## Discussion

A set of interventions were developed, refined and implemented with the aim of reducing prescribing errors made by junior doctors. Process measures and qualitative findings indicate some benefits. Although the net controlled effects of the interventions were estimated decreases in both trend and level of weekly prescribing error rates at the intervention site, these did not reach statistical significance.

### Relationship to other evidence

Our findings are in line with a Cochrane review and more recent update,^7^ ^19^ which suggest that providing feedback results in small-to-moderate positive effects on professional practice and that process measures may be more sensitive to feedback initiatives than outcome measures. However, these reviews focus on studies of feedback on specific aspects of clinical practice, such as prescribing for a particular clinical condition; no studies of hospital prescribing errors were included. Our prescribing error rate was comparable with UK studies employing similar methodologies.^2^

### Strengths and limitations

In designing an evaluation based on mixed methods including a controlled interrupted time series analysis, we have applied a robust quasi-experimental design to a quality improvement initiative; such initiatives are rarely subjected to this level of evaluation. We were therefore able to examine any change in prescribing error rates above and beyond those predicted by a baseline trend which might, for example, describe natural improvement in junior doctors' prescribing as they gain experience. Importantly, the survey and qualitative components of this study allowed for generation of insights into the processes involved and maximisation of the transferable value of the study. The questionnaire had a good response rate and the junior doctor focus group was well attended—we believe the findings to be representative of the cohort. Since only four pharmacists participated in their focus group, these results may represent a more specific set of views.

The main limitation was that the new junior doctor intake was contemporaneous with the initiation of our interventions at site 1, potentially confounding detection of any effect of our intervention on error rates. A longer pre-intervention baseline at both sites would have been preferable. These issues were mainly due to the dates for both project-funding and junior doctors' inductions being fixed. Other limitations were that senior pharmacists were only able to assess pharmacists' feedback provision on five occasions, and we recognise that pharmacists collecting prescribing error data may vary in their adherence to data collection procedures. Finally, we provided fortnightly run charts to the junior doctors showing prescriber identification rates as we were collecting these data as part of our evaluation. However this relied on manual data collection and was quite time-consuming and so is unlikely to be sustainable in routine practice.

### Interpretation

We achieved our first objective of improving prescriber identification, although it appears that the percentage of junior doctors' medication orders for which the prescriber could be identified hit a ceiling of around 50%. Possible reasons for this are that name stamps were lost or forgotten and for some sections of the medication chart the signature box is very small. It is also difficult to depress the stamp onto the chart without resting it on a firm surface (a particular problem on ward rounds)*.* Introducing a change where junior staff may differ in practice to their senior role models is also likely to be difficult. We suspect that it will not be possible to achieve 100% prescriber identification until inpatient electronic prescribing is introduced. Routine use of signature logs could form part of a solution in the meantime. We also achieved our second objective of providing effective feedback to prescribers at both the individual level and group level, with our interventions well received by both pharmacists and junior doctors.

Critical analysis of our logic model (figure 1) suggests why we may not have detected a significant overall reduction in prescribing error rates. The model is based on the assumption that increasing prescriber identification would lead to increased feedback by pharmacists, leading to changes in prescribing behaviour and a reduction in prescribing error rates. A feedback initiative of this type therefore relies on reliable identification of potential ‘learners’, opportunities for the timely communication of relevant and credible feedback, interaction with individual psychology to produce ‘learning’ and an environment conducive with implementing that learning in practice. Our work highlights the need for a more comprehensive theory of change in this area, underpinned by broad synthesis of theory and experience across a range of application contexts, to understand how contextual and intervention factors affect the success of feedback initiatives. For example, we were only able to increase the identification of junior doctor prescribers to about 50%. Although a substantial increase from baseline, this meant that the prescriber still could not be identified for 50% of medication orders, thus limiting opportunities for individual feedback. We did not set out to assess the percentage of junior doctors' prescribing errors for which a pharmacist gave individual feedback, but assume this would not be 100% as time constraints, shift patterns and individual motivation are likely to prevent some opportunities for feedback.

Prescribing errors are multifactorial^2^ ^4^ ^5^ ^25^ ^26^ and it is likely that feedback would only prevent a subset, especially if feedback relates to drugs that are rarely prescribed. One of our most common error types related to medication reconciliation on admission, which may be largely a system problem and less ameliorable through individual feedback. Detection of effects of feedback on error rates is likely to require sustained monitoring over longer time periods, with an adequate baseline, comprehensive monitoring of compliance with the intervention and stable participant groups over baseline and intervention periods. Based on our experience here, we would recommend at least 3 months' weekly error rate data be collected both pre-intervention and post-intervention, with quasi-experimental control over potential confounders (such as changes in junior doctor cohort) through careful selection of a comparable control site. These methodological requirements may represent a significant challenge, but our study suggests that effective feedback on prescribing errors has the potential to support beneficial learning. The impact on prescribing errors, ideally in combination with other interventions, therefore warrants further investigation. Further work should also explore how information technology could be used to deliver individualised feedback around prescribing errors in the hospital setting.

#### Lessons learnt from the project's evolution

We identified a number of learning points from our PDSA cycles. We found that the names some doctors used in practice differed from their given names (such as use of their middle name rather than their first name) and we subsequently attempted to establish and use preferred names on the name stamps. Some doctors initially believed that the stamp replaced a prescribing signature; we clarified that a signature was required alongside a stamp. The PDSA approach allowed us to identify and address such issues early on.

Locally, we now issue name stamps to all junior doctors and non-medical prescribers. We audit prescriber identification annually and continue to send out fortnightly prescribing tips. The requirement of providing feedback to the initial prescriber has been incorporated into pharmacy standard operating procedures. Our interventions were subsequently introduced at two further large teaching hospitals within a different organisation and are now being rolled out across North West London.

## Conclusion

Using a set of three linked interventions, we increased the percentage of medication orders for which junior doctor prescribers stated their name from about 10% to 50%. Principles of effective feedback were developed in conjunction with junior doctors and both quantitative and qualitative evaluation suggested an improvement in experiences around feedback. No change in prescribing error prevalence was detected. Attempts to produce a measureable reduction in prescribing errors are likely to need a multifaceted approach of which feedback should form part.
