# Supplementary material for: Improving feedback on junior doctors’ prescribing errors: mixed-methods evaluation of a quality improvement project
Source: BMJ Qual Saf. 2016 Apr 4;26(3):240–7. doi: 10.1136/bmjqs-2015-004717 (PMC5339559; doi:10.1136/bmjqs-2015-004717)
Supplement: Supplementary appendix 1 [file bmjqs-2015-004717supp_appendix1.pdf]

Appendix S1: UK hospital doctor grades

Changes were made to medical graduates' training in 2005. A two year foundation programme replaced the one-year pre-registration house officer term and the first year of the previous two-year senior house officer term. The current terms in use are as follows:

Foundation Year 1 (FY1)

These are doctors in their first year of practice following completion of their medical degree. Graduates are only provisionally registered with the General Medical Council (GMC) for the first year. On successfully completing their first year they become fully registered with the GMC. These doctors were previously known as house officers, or pre-registration house officers.

Foundation Year 2 (FY2)

These are doctors in their second year of practice, previously known as year 1 senior house officers.

Specialist Registrars

These are doctors training in their chosen speciality; the years in training depends on the type of speciality, but on completion of their training doctors are awarded a Certificate of Completion of Training and are entitled to enter the Specialist Register or the General Practitioner Register.

Consultant

These are the most senior doctors within their own speciality; they usually work with a team of more junior doctors. The consultant is ultimately responsible for the patients who are referred to their care.
