# Supplementary material for: Improving feedback on junior doctors’ prescribing errors: mixed-methods evaluation of a quality improvement project
Source: BMJ Qual Saf. 2016 Apr 4;26(3):240–7. doi: 10.1136/bmjqs-2015-004717 (PMC5339559; doi:10.1136/bmjqs-2015-004717)
Supplement: Supplementary appendix 2 [file bmjqs-2015-004717supp_appendix2.pdf]

Appendix S2: examples of prescribing tips

Avoid errors when prescribing IV Pabrinex®: Good Prescribing Tip of the fortnight

(this prescription has been re-written to maintain the anonymity of the prescriber)

This is a prescription for an adult patient, what is the dose for this prescription?

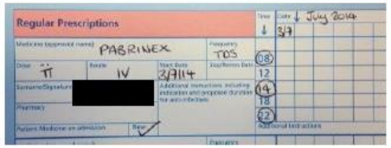

a) 1 pair ('ampoule I' + 'ampoule II') TDS
b) 2 pairs i.e. 2 x ('ampoule I' + 'ampoule II') TDS
c) Only 'ampoule II' TDS
d) Not sure

Answer: it's not clear!

The error:

The intended prescription is for 2 pairs of Pabrinex® ('ampoule I' + 'ampoule II') three times a day. However, the prescription can be easily misinterpreted.

The clearest way to prescribe the intended dose is:
Medicine (approved name): **Pabrinex I + II** Dose: **2 pairs** Frequency: **TDS**

Ambiguous prescribing of Pabrinex® may lead to the inappropriate treatment of Wernicke-Korsakoff Syndrome which can have fatal consequences

Prescribing tips:

- One single dose of Pabrinex® consists of both **ampoule I AND ampoule II**

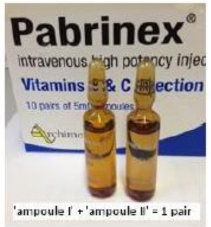

- Both ampoules must be administered:
  - **Ampoule No. I** contains **thiamine, riboflavin** and **pyridoxine**
  - **Ampoule No. II** contains **ascorbic acid, nicotinamide** and **anhydrous glucose**
- Pabrinex® should be prescribed as either '**1 pair**' or '**2 pairs**' so the instruction for administration is clear:

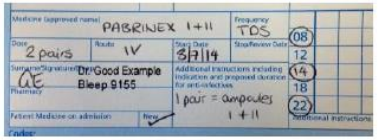

Remember to use your name-stamp or print your name when prescribing

Anonymised for review purposes

Avoid errors when prescribing drugs with unusual frequencies: Good Prescribing Tip of the fortnight

(this prescription has been re-written to maintain the anonymity of the prescriber)

This is a prescription for an adult patient, can you spot the error?

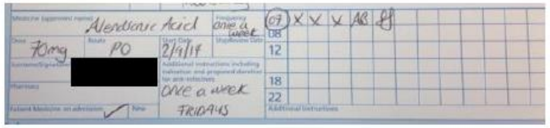

The error:

This patient erroneously received alendronic acid (alendronate) on **two consecutive days** instead of once a week because the dose administration section of the chart had not been clearly marked.

An overdose of alendronic acid may cause hypocalcaemia, hypophosphataemia or upper gastro-intestinal adverse events

For drugs with an unusual dosing schedule, the days and times the dose is due must be indicated explicitly on the administration section of the drug chart, otherwise the patient may receive an incorrect dose

Prescribing tips:

- Explicitly indicate when the **dose is due** by **marking** the whole of the dose administration section of the chart, to ensure the drug is administered on the **intended days** and **times** only.
- Some examples of prescribing drugs with unusual frequencies:

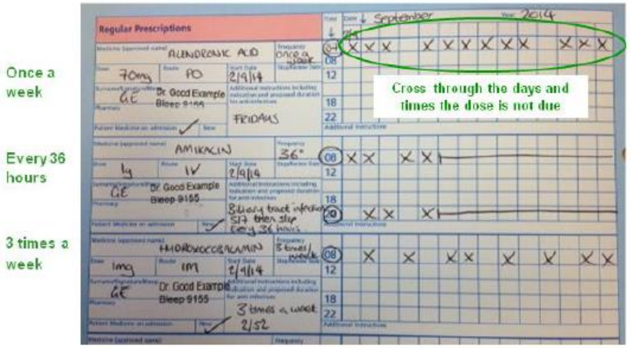

Remember to use your name-stamp or print your name when prescribing

Anonymised for review purposes
