# Supplementary material for: Improving feedback on junior doctors’ prescribing errors: mixed-methods evaluation of a quality improvement project
Source: BMJ Qual Saf. 2016 Apr 4;26(3):240–7. doi: 10.1136/bmjqs-2015-004717 (PMC5339559; doi:10.1136/bmjqs-2015-004717)
Supplement: Supplementary appendix 3 [file bmjqs-2015-004717supp_appendix3.pdf]

Appendix S3: Questionnaires

The Prescribing Improvement Model
Prescribing Questionnaire for Foundation Year 1 Doctors

This questionnaire is part of a study helping us improve how pharmacists give feedback on prescribing errors to prescribers: your views on prescribing errors are **important**.

Current site: Gender: Male ☐; Female ☐

Previous sites worked at: Undergraduate medical school:

Please circle the number which best represents your agreement with the statement. Replies are anonymous.

	Understanding prescribing errors	Strongly disagree							Strongly agree
1.	I feel there is an open culture in this organisation with respect to discussing prescribing errors.	1	2	3	4	5	6	7	8
2.	I understand why doctors make prescribing errors	1	2	3	4	5	6	7	8
3.	When I have made prescribing errors in the past, I understand why I made them	1	2	3	4	5	6	7	8
4.	I am aware of the conditions under which I am likely to make a prescribing error	1	2	3	4	5	6	7	8
5.	I believe I am aware of all major prescribing errors I make	1	2	3	4	5	6	7	8
6.	I believe I am aware of all minor prescribing errors I make	1	2	3	4	5	6	7	8
7.	I think knowing about the prescribing errors I make is important	1	2	3	4	5	6	7	8
8.	I think knowing about the prescribing errors others make is important	1	2	3	4	5	6	7	8

	Evaluation of the current feedback you receive from pharmacists on prescribing errors	Strongly disagree							Strongly agree
9.	The feedback I currently receive is useful in improving my prescribing	1	2	3	4	5	6	7	8
10	I feel I receive verbal feedback often enough for it to be useful	1	2	3	4	5	6	7	8
11	I feel I receive written feedback often enough for it to be useful	1	2	3	4	5	6	7	8
12	I receive verbal feedback soon enough after the event to be useful	1	2	3	4	5	6	7	8
13	I receive written feedback soon enough after the event to be useful	1	2	3	4	5	6	7	8
14	The feedback I receive on prescribing errors is highly relevant to my personal practice	1	2	3	4	5	6	7	8
15	I believe that the information I receive on prescribing errors from pharmacists is accurate	1	2	3	4	5	6	7	8
16	I believe that the information I receive on prescribing errors is from a trusted source	1	2	3	4	5	6	7	8
17	The feedback I receive from pharmacists on prescribing errors is provided in a constructive manner	1	2	3	4	5	6	7	8
18	Receiving feedback is a valuable use of my time	1	2	3	4	5	6	7	8

Please continue overleaf

	How is pharmacists' feedback on prescribing errors received?	Strongly disagree							Strongly agree
19.	I am always receptive to feedback I receive from pharmacists on my prescribing errors	1	2	3	4	5	6	7	8
20.	The feedback I receive from pharmacists on prescribing errors is non-threatening	1	2	3	4	5	6	7	8

	Response to receiving feedback on prescribing errors from pharmacists	Strongly disagree							Strongly agree
21	Receiving feedback on prescribing errors has caused me to reflect on my prescribing practice	1	2	3	4	5	6	7	8
22	I have made changes to my prescribing practice in response to feedback received from pharmacists	1	2	3	4	5	6	7	8
23	I am happy to discuss prescribing errors with my peers	1	2	3	4	5	6	7	8
24	I think receiving formal feedback on prescribing errors means I would make fewer errors in the future	1	2	3	4	5	6	7	8

	What kind of prescribing error feedback would you like to receive?	Strongly disagree							Strongly agree
25	I want to be told of all major prescribing errors I make	1	2	3	4	5	6	7	8
26.	I want to be told of all prescribing errors I make, however minor	1	2	3	4	5	6	7	8
27.	I would like to have feedback by pharmacists which includes examples of specific errors I make	1	2	3	4	5	6	7	8
28	Feedback by pharmacists which includes generic guidance on prescribing practice would be useful	1	2	3	4	5	6	7	8
29	Feedback which includes statistical comparison with my peers would be useful	1	2	3	4	5	6	7	8
30	I prefer to receive feedback on prescribing errors from pharmacists rather than senior doctors	1	2	3	4	5	6	7	8

Do you have any comments or suggestions that would help pharmacists to provide better feedback?

What is the best format for pharmacists to provide feedback on prescribing errors to you? Consider mode of delivery, content, format, frequency etc.

The Prescribing Improvement Model Questionnaire for Pharmacists

We want to support safe prescribing, increase doctors' **confidence**, enhance **learning** and make pharmacists' feedback more **effective** - your views are **important**.

Please only complete this questionnaire if you provide a ward pharmacy service

Which site do you most often provide **weekday** ward cover on: 1st______; 2nd______; 3rd______.

Gender: Male ☐; Female ☐ Band: 6 ☐; 7 ☐; 8 ☐

Please circle the number which best represents your agreement with the statement. Replies are anonymous.

Understanding prescribing errors	Strongly disagree							Strongly agree
1. I feel there is an open culture with respect to discussing prescribing errors	1	2	3	4	5	6	7	8
2. I understand why doctors make prescribing errors	1	2	3	4	5	6	7	8
3. I am aware of the conditions under which doctors are likely to make a prescribing error	1	2	3	4	5	6	7	8
4. I believe FY1s are aware of all **major** prescribing errors they make	1	2	3	4	5	6	7	8
5. I believe FY1s are aware of all **minor** prescribing errors they make	1	2	3	4	5	6	7	8
6. I think it is important that FY1s know about prescribing errors **other FY1s** make	1	2	3	4	5	6	7	8
**Evaluation of the current feedback FY1s receive from pharmacists on their prescribing errors**	Strongly disagree							Strongly agree
7. In this trust pharmacy support FY1s in learning from their prescribing errors	1	2	3	4	5	6	7	8
8. Robust processes are in place in this trust for monitoring and feeding back information about prescribing errors	1	2	3	4	5	6	7	8
9. When I identify a prescribing error I always make a doctor aware that an error has been made	1	2	3	4	5	6	7	8
10. The feedback I currently give is useful in improving FY1s' prescribing	1	2	3	4	5	6	7	8
11. I am able to give **verbal** feedback **often** enough for it to be useful to FY1s	1	2	3	4	5	6	7	8
12. I am able to give **written** feedback (e.g. in medical notes) **often** enough for it to be useful to FY1s	1	2	3	4	5	6	7	8
13. I am able to give **verbal** feedback to FY1s **soon** enough after detecting an error for it to be useful	1	2	3	4	5	6	7	8
14. I am able to give **written** feedback (e.g. in medical notes) to FY1s **soon** enough after detecting an error for it to be useful	1	2	3	4	5	6	7	8

PLEASE CONTINUE OVERLEAF

15. I believe that the information FY1s receive on prescribing errors is accurate	1	2	3	4	5	6	7	8
16. I believe that FY1s find the feedback they receive on prescribing errors to be trustworthy	1	2	3	4	5	6	7	8
17. I believe that I provide feedback to FY1s on prescribing errors in a constructive manner	1	2	3	4	5	6	7	8
18. I always identify the specific prescriber who makes a prescribing error	1	2	3	4	5	6	7	8
19. Whenever I identify a prescribing error I give feedback to the specific prescriber who made the error	1	2	3	4	5	6	7	8
20. I feel comfortable talking to FY1s about prescribing errors	1	2	3	4	5	6	7	8
21. I feel comfortable informing FY1s they have made a prescribing error	1	2	3	4	5	6	7	8
22. Giving feedback is a valuable use of pharmacists' time	1	2	3	4	5	6	7	8
**How is pharmacists' feedback on prescribing errors received by doctors?**	Strongly disagree							Strongly agree
23. FY1s are always interested in and engaged with the feedback they receive on their prescribing errors	1	2	3	4	5	6	7	8
24. I believe that FY1s find the feedback they receive to be non-threatening	1	2	3	4	5	6	7	8
**Support for giving feedback on prescribing errors to FY1s**	Strongly disagree							Strongly agree
25. I feel supported by my organisation to give feedback to FY1s on their prescribing errors	1	2	3	4	5	6	7	8
**What kind of prescribing error feedback would you like to give to the FY1s?**	Strongly disagree							Strongly agree
26. I want to inform FY1s of all **major** prescribing errors they make	1	2	3	4	5	6	7	8
27. I want to inform FY1s of **all** prescribing errors they make, however minor	1	2	3	4	5	6	7	8
28. I would like to give feedback to individual FY1s which includes examples of specific errors they have personally made	1	2	3	4	5	6	7	8
29. Feedback to FY1s which includes generic guidance on prescribing practice would be useful	1	2	3	4	5	6	7	8
30. Feedback to FY1s which includes statistical comparison with their peers would be useful	1	2	3	4	5	6	7	8

Do you have any comments or suggestions that would help pharmacists to provide better feedback?

____

____

What is the best format for pharmacists to provide feedback to FY1s on prescribing errors? Consider mode of delivery, content, format, frequency etc.

____

____
