# Supplementary material for: Improving feedback on junior doctors’ prescribing errors: mixed-methods evaluation of a quality improvement project
Source: BMJ Qual Saf. 2016 Apr 4;26(3):240–7. doi: 10.1136/bmjqs-2015-004717 (PMC5339559; doi:10.1136/bmjqs-2015-004717)
Supplement: Supplementary appendix 4 [file bmjqs-2015-004717supp_appendix4.pdf]

Appendix S4: Perceptions of feedback effectiveness (longitudinal data from site 1 for items included in the overall feedback effectiveness scale; significant effects shaded)

Table 1: pharmacists

	Timepoint								
	1 (Spring 2013)				2 (Spring 2014)				
	Mean	Standard Deviation	Median	Range	Mean	Standard Deviation	Median	Range	Change in Mean
09 The feedback I currently give is useful in improving FY1s' prescribing	5.68_a	1.77	6	8	6.56_a	1.50	7	6	0.88
10 I am able to give verbal feedback often enough for it to be useful to FY1s	5.08_a	1.53	5	4	5.17_a	2.09	6	7	0.09
11 I am able to give written feedback (e.g. in medical notes) often enough for it to be useful to FY1s	3.56_a	1.85	3	6	3.06_a	1.51	4	5	-0.50
12 I am able to give verbal feedback to FY1s soon enough after detecting an error for it to be useful	5.44_a	1.58	6	6	5.67_a	1.37	6	6	0.23
13 I am able to give written feedback (e.g. in medical notes) to FY1s soon enough after detecting an error for it to be useful	3.80_a	2.06	3	6	3.33_a	1.75	4	6	-0.47
15 I believe that the information FY1s receive on prescribing errors is accurate	5.32_a	1.63	6	7	6.72_b	1.07	7	4	1.40
16 I believe that FY1s find the feedback they receive on prescribing errors to be trustworthy	5.80_a	1.04	6	5	6.67_b	0.91	7	3	0.87

17 I believe that I provide feedback to FY1s on prescribing errors in a constructive manner	6.16_a	0.99	6	4	6.83_a	1.20	7	5	0.67
18 Giving feedback is a valuable use of pharmacists' time	6.72_a	1.17	7	5	7.22_a	1.26	8	5	0.50
31 In this trust pharmacy support FY1s in learning from their prescribing errors	5.04_a	1.67	6	6	6.22_a	1.63	6	6	1.18
32 Robust processes are in place in this trust for monitoring and feeding back information about prescribing errors	4.56_a	1.56	5	6	5.44_a	1.46	6	5	0.88
33 When I identify a prescribing error I always make a doctor aware that an error has been made	5.60_a	1.63	6	6	6.50_a	0.79	6	3	0.90
34 I always identify the specific prescriber who makes a prescribing error	4.24_a	1.83	4	6	4.83_a	2.15	5	7	0.59
35 Whenever I identify a prescribing error I give feedback to the specific prescriber who made the error	4.04_a	1.74	4	6	5.28_a	1.84	6	6	1.24
36 I feel comfortable talking to FY1s about prescribing errors	6.60_a	1.26	7	4	7.00_a	0.69	7	2	0.40
37 I feel comfortable informing FY1s they have made a prescribing error	6.48_a	1.33	7	4	6.94_a	0.64	7	2	0.46
Pharm.Eval	5.26_a	.88	5	3	5.84_b	0.80	6	3	0.58

Notes: Values in the same row and sub-table not sharing the same subscript are significantly different in the two-sided test of equality for column means (items evaluated at $p<0.01$; Evaluative scale score evaluated at $p<0.05$). Cells with no subscript are not included in the test. Tests assume equal variances.

Table 1: Foundation year 1 doctors

	Timepoint								
	1 (Spring 2013)				2 (Spring 2014)				
	Mean	Standard Deviation	Median	Range	Mean	Standard Deviation	Median	Range	Change in Mean
09 The feedback I currently receive is useful in improving my prescribing	6.35_a	1.13	6	4	6.68_a	0.95	7	3	0.33
10 I feel I receive verbal feedback often enough for it to be useful	5.92_a	1.65	6	6	6.11_a	2.02	7	7	0.19
11 I feel I receive written feedback often enough for it to be useful	3.58_a	1.72	4	6	4.84_a	1.86	5	7	1.26
12 I receive verbal feedback soon enough after the event to be useful	5.81_a	1.55	6	6	5.74_a	2.10	6	7	-0.07
13 I receive written feedback soon enough after the event to be useful	4.15_a	2.07	4	7	4.16_a	1.74	4	6	0.01
14 The feedback I receive on prescribing errors is highly relevant to my personal practice	6.58_a	0.95	7	3	6.74_a	1.19	7	4	0.16
15 I believe that the information I receive on	7.19_a	.69	7	2	7.00_a	0.82	7	3	-0.19

prescribing errors from pharmacists is accurate									
16 I believe that the information I receive on prescribing errors is from a trusted source	7.23_a	0.86	7	3	6.95_a	0.85	7	3	-0.28
17 The feedback I receive from pharmacists on prescribing errors is provided in a constructive manner	7.36_a	0.81	8	2	7.05_a	0.97	7	3	-0.31
18 Receiving feedback is a valuable use of my time	7.35_a	.80	8	2	7.00_a	1.11	7	4	-0.35
FY1.Eval	6.15_a	.74	6	3	6.23_a	.86	6	4	0.08

Note: Values in the same row and sub-table not sharing the same subscript are significantly different in the two-sided test of equality for column means (items evaluated at $p<0.01$; Evaluative scale score evaluated at $p<0.05$). Cells with no subscript are not included in the test. Tests assume equal variances.
