# Supplementary material for: Improving feedback on junior doctors’ prescribing errors: mixed-methods evaluation of a quality improvement project
Source: BMJ Qual Saf. 2016 Apr 4;26(3):240–7. doi: 10.1136/bmjqs-2015-004717 (PMC5339559; doi:10.1136/bmjqs-2015-004717)
Supplement: Supplementary appendix 5 [file bmjqs-2015-004717supp_appendix5.pdf]

Appendix S5: Types of errors identified

Technical Errors			Clinical Errors		
**Error description**	**Site 1**	**Site 2**	**Error description**	**Site 1**	**Site 2**
Missing drug name	0	0	Incorrect drug	15	15
Missing dose	5	14	Incorrect dose	96	79
Missing duration of treatment	2	1	Incorrect duration of therapy	5	11
Missing frequency or dosing schedule	10	7	Incorrect frequency or dosing schedule (but correct daily dose)	22	15
Missing route	7	0	Incorrect route	9	3
Missing formulation or brand name where relevant or required	6	4	Incorrect formulation	20	27
Missing signature	15	31	Medication omitted when clinically indicated	67	105
Missing date	22	9	Drug prescribed is not indicated for patient	26	8
Missing patient information	0	1	Failure to take into account a drug interaction	2	4
Incorrect patient information	0	0	Drug prescribed is contra-indicated	6	0
Inappropriate abbreviation	3	8	Duplicated therapy	20	21
Illegible or unclear prescription	1	2	Prescribing a drug to which the patient is allergic	1	1
Missing instructions for use or administration	0	4	Incorrect instructions for use or administration	5	7
Missing stop or review date for antibiotic therapy	13	7			
Missing indication stated for antibiotic therapy	11	6			
Incorrect spelling of drug name (excludes minor misspelling of a drug name)	1	1			
**Technical total**	**96**	**95**	**Clinical total**	**294**	**296**
